# The impact of childhood sexual abuse and childhood traumatic events on outcome in adult inpatients with eating disorders

**DOI:** 10.1186/s40337-025-01316-9

**Published:** 2025-06-19

**Authors:** Siri Weider, Øyvind Rø, Sigrid Bjørnelv, Marit Danielsen

**Affiliations:** 1 Department of Psychology, The Norwegian University of Science and Technology (NTNU), Trondheim, Norway; 2 Eating Disorder Unit, Department of Psychiatry, Levanger Hospital, Nord-Trøndelag Hospital Trust, Levanger, Norway; 3 Regional Department for Eating Disorders, Division of Mental Health and Addiction, Oslo University Hospital, Oslo, Norway; 4 Institute of Clinical Medicine, Division of Mental Health and Addiction, University of Oslo, Oslo, Norway

**Keywords:** Anorexia nervosa, Bulimia nervosa, Binge eating disorder, Other specified feeding and eating disorders, Childhood sexual abuse, Childhood trauma events, Remission rates

## Abstract

**Background:**

Childhood traumatic events (CTE) are frequently described in patients with eating disorders. However, the understanding of how such events impact eating disorder treatment outcome is limited. The aim of this study was to examine the prevalence of childhood sexual abuse (CSA) or any CTE at baseline in a naturalistic transdiagnostic sample, and to evaluate how such events affect symptom change and rates of remission at follow-up.

**Methods:**

The sample comprised 228 adult female former eating disorder inpatients (M_age_ = 24.6 years), of which 61.4% (*n* = 140) had been diagnosed with anorexia nervosa at baseline, 21.1% (*n* = 48) with bulimia nervosa, and 17.5% (*n* = 40) with other specified feeding or eating disorder including binge eating. Data on CSA/ CTE exposure were collected from the patients’ hospital records and were rated for degree of severity (severe, moderate to low, or no). Analyses of prevalence, group differences, and rates of remission at follow-up were performed.

**Results:**

Findings showed high prevalence of high severity CSA and CTE at admission, respectively 33% (*n* = 75) and 48.7% (*n* = 111). Moreover, although all patients showed significant improvement in symptoms from baseline to follow-up, a significant association was found between severity of CTE exposure and remission group affiliation with 24% of those with severe CTE exposure and 40% of those with no CTE exposure being in remission.

**Conclusions:**

Despite considerable heterogeneity in demographic characteristics, treatment and length of follow-up, severity of CTE exposure was associated with remission at follow-up. Thus, patients with the highest CTE severity showed the poorest prognoses. Findings from this study underline the importance of addressing trauma experiences in assessment and therapy and indicate that CTE severity level should be considered when assessing trauma experiences.

## Abstract (plain language summary)

In a group of patients with different types of eating disorder, high prevalences of childhood traumatic events were found. Patients that had experienced severe childhood sexual abuse had more eating disorder- and depressive symptoms at baseline than patients without such events. In addition, patients that had experienced any severe childhood traumatic event had slightly higher eating disorder symptoms than those without such events and than those that had experienced less severe trauamtic events. Although patients with eating disorders and childhood traumatic events had significantly less symptoms at follow-up than at baseline, those with more severe traumatic events had lower levels of remission than patients without traumatic experiences. This underlines the importance of addressing trauma events in eating disorder treatment.

## Background

Despite extensive research on the effectiveness of different therapeutic interventions for eating disorder (ED) patients, the long-term outcome is still unsatisfactory [1, 2]. There is therefore an urgent need to identify and develop differentiated treatment options for patients with EDs and different comorbid illnesses or symptom profiles, especially for subgroups that in clinical practice have shown poor outcome.

### Associations between traumatic events and EDs

A substantial number of patients with EDs report experiences of childhood traumatic events (CTE) [3]. In a nationally representative sample from the US, individuals with EDs were found to be more likely to have faced childhood maltreatment (CM; defined as harsh physical punishment, physical-, sexual-, or emotional abuse, emotional- or physical neglect, and exposure to intimate partner violence) than the general population [4]. Moreover, in a systematic review and meta-analysis, life-time prevalence of CM (defined as physical-, sexual-, or emotional maltreatment, neglect or exploitation) in patients with EDs was found to be 21–59% compared to 1–35% and 5–46% in healthy controls and psychiatric controls, respectively [5]. Also, in a recent Norwegian study investigating patients at 17 years follow-up, 71% of the patients reported experiencing at least one type of CM, including 38.7% childhood sexual abuse (CSA) [6]. Such events have been linked to the development of a range of mental health problems [7, 8], and in a recent study, adolescents that had faced childhood adverse experiences (CM and/or household dysfunction such as household substance abuse, mental illness or witnessing violence etc.) were evaluated to be at a higher risk of developing an ED, a risk that exceeded the burden from family dysfunction [9].

### The importance of the different types of childhood traumatic events

Associations have been found between EDs and different types of traumatic events such as emotional abuse/neglect [5, 9–11], physical abuse/violence [5, 9, 11], and CSA [5, 9, 12, 13]. Among these types, CSA has been of special interest, and the development of an ED has been suggested as a maladaptive means to regulate emotional suffering or as a method to “clean” off such trauma experiences. Furthermore, other types of childhood adverse events including peer victimization/bullying have also been associated with EDs [14]. Interestingly, in an umbrella review investigating a total of 50 different risk factors for EDs in a sample of almost 30,000 patients with EDs, evidence for the associations were classified as convincing, highly suggestive, suggestive or weak. No risk factor was found to be convincingly supported. However, the associations between appearance-related victimization and any ED, and between CSA and bulimia nervosa (BN), were found to be supported by highly suggestive evidence, underscoring the importance of victimization as a potential risk factor for EDs [15].

Previous findings suggest that comorbid EDs and traumatic event exposure are associated with more severe symptoms as well as increased levels of psychiatric comorbidity, suicidal ideation, and self-harm behavior than EDs without such comorbidity [5], and there is support for a dose-response relationship between trauma frequency and EDs. For instance, in a large Australian cohort study, it was found that the incidence of partial BN, defined as meeting two criteria for the full EDs, was associated with CSA, and for those reporting two or more episodes of CSA, there was a 5-fold increase in new incidents of partial BN [16]. In patients with partial anorexia nervosa (AN), however, there were lower incidents of CSA, and no association was found. A dose-response relationship has also been observed by others [5, 17], indicating a cumulative effect of trauma exposure.

### Trauma exposure and treatment outcome

Whether the experience of traumatic events impacts treatment outcome has been subject to some controversy. Some studies have failed to find any difference in outcome between patients with EDs with or without traumatic experiences. For instance, in a study on treatment outcome after intensive enhanced cognitive behavioral therapy for adults with AN, no difference was found between patients with or without CSA [18]. However, more patients with childhood adverse events were found to drop out of treatment after Cognitive Behavioral Therapy (CBT) compared to patients without childhood adverse experiences [19]. Additionally, at 3-year follow-up, only 12.1% of the patients with EDs with childhood adverse events had reached full recovery from any Axis 1 diagnosis compared to 31.0% of patients with EDs without childhood adverse events [19]. Although the persistence of psychopathology was mainly attributable to diagnoses other than the ED, the authors point to the importance of considering the complexity of these patients’ symptomatology, and the limitations of CBT as a stand-alone treatment. Furthermore, Anderson, LaPorte et al. [20], found that inpatients with BN with a history of CSA displayed more symptoms at treatment initiation, at all weekly assessments and at three months post discharge compared to patients with BN with no history of CSA. Although both groups in the abovementioned study showed a decreased level of symptoms post treatment, the individuals with BN and CSA were more likely to be readmitted to inpatient care. Also, in a study on treatment outcome of patients with binge eating disorder (BED) and CM exposure, the results showed that both childhood abuse and posttraumatic stress disorder (PTSD) predicted poorer treatment outcome. However, childhood abuse predicted poorer outcome only in patients with comorbid PTSD [21].

In a systematic review on the association between PTSD, traumatic experiences and ED treatment outcome, Convertino and Mendoza [22] observed that patients that had experienced CTE were more likely to drop out of treatment than patients without such experiences. Furthermore, although no difference was found between patients with or without CTE exposure in reduction in ED symptoms from treatment, there were indications of greater relapse for patients having been exposed to traumatic events [22].

Additionally, in the above-mentioned study by Eielsen et al. [6], the authors investigated different change trajectories in patients with EDs 17 years after inpatient treatment. They identified four different classes, defined by the change in the global score on the Eating Disorder Examination interview, and found that those who had been exposed to CSA were overrepresented in the two classes indicating poorer long-term outcome [6].

A few studies have investigated the significance of dose/frequency and severity of childhood traumatic events and treatment outcome. Notably, a dose-effect association between childhood trauma and premature termination of outpatient treatment in patients with BN has been observed [23]. However, in the aforementioned study by Anderson et al. [20], an evaluation of severity was completed, but contrary to expectations, no difference in treatment outcome between the three severity level groups were found.

### Aims of the study

Based on the findings above and the scarcity of research on outcome after treatment for patients with EDs and trauma exposure, we investigated data from a series of transdiagnostic female patients with EDs with a wide variety of comorbidities, age, body mass index (BMI), length of illness, length of treatment and length of follow-up, in an inpatient setting over a period of almost 20 years. In doing so, we chose to investigate the significance of any CTE including CSA. In addition, based on a hypothesis that CSA might be most strongly associated with EDs, we also wanted to investigate this type of trauma separately. The aims were to evaluate the prevalence of any CTE and/or CSA, to examine if there were any differences between patients with or without CTE and/or CSA exposure in self-reported symptoms at baseline, to investigate how CTE and/or CSA affects symptom change at follow-up and to evaluate how CTE and/or CSA affects rates of remission at follow-up.

## Methods

### Participants and procedure

**Fig. 1:**
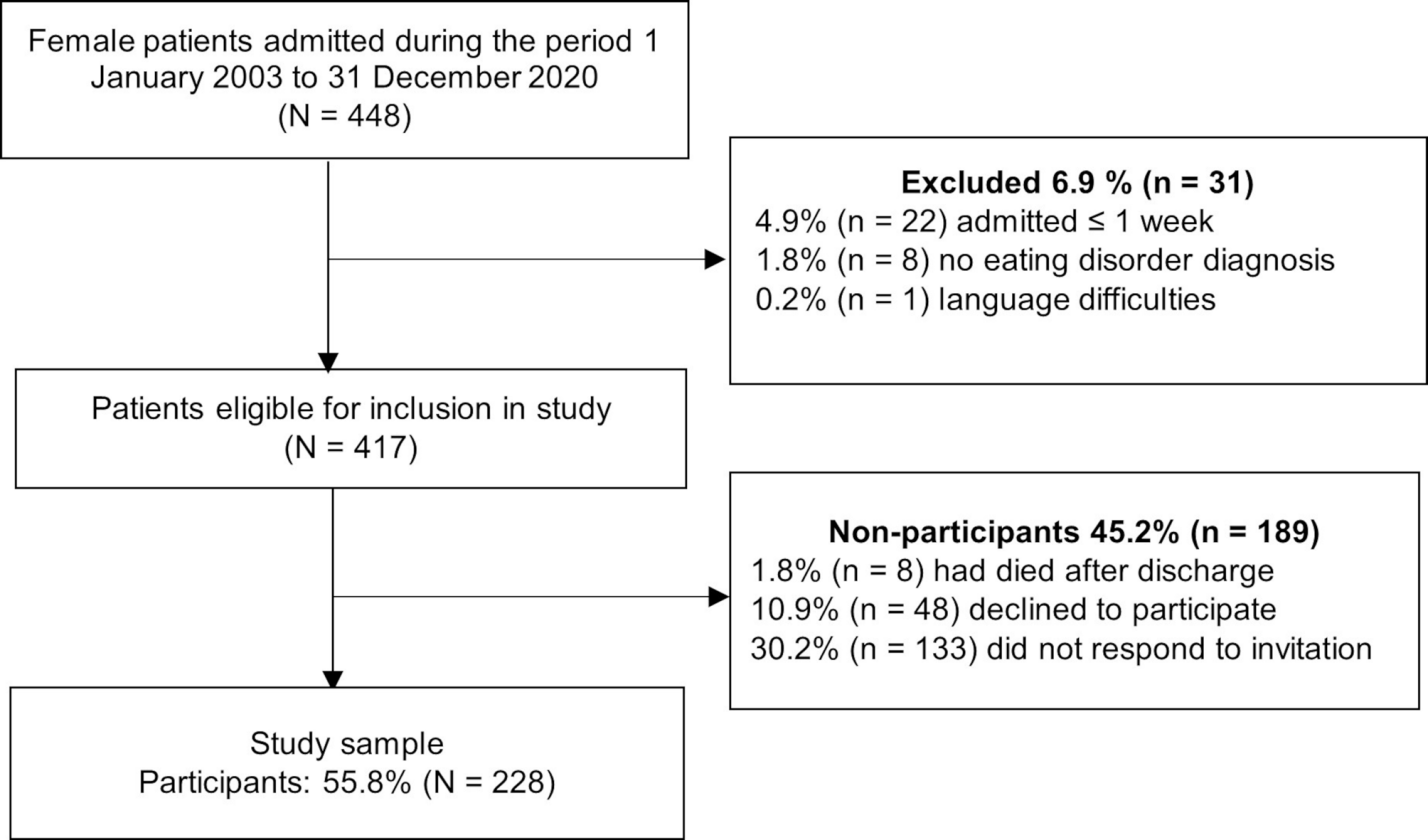
Flowchart of the study sample

Between January 1st, 2003, and December 31st, 2020, a total of 448 female patients were admitted to the ED inpatient unit at Levanger Hospital. All had previously been treated at the primary (municipal health care service) and/or secondary (specialist e.g. hospital) health level without satisfactory treatment outcome and were evaluated to be medically stable for psychiatric treatment. From 2009 onwards, former patients were invited to participate in a study on the trajectories of EDs. If the patient consented to this, data from their admission and follow-up data could be included in the study. Inclusion criteria were fulfilling the criteria for any ED and receiving the specialized inpatient treatment program at the unit. In addition, being able to complete questionnaires in Norwegian was necessary. Exclusion criteria were severe medical instability (*n* = 0). In addition, for the present purpose, males were excluded due to the low frequency (*n* = 7). Thus, 417 patients were eligible to participate. Among these, 45.2% (*n* = 189) were non-participants, leaving a study sample consisting of 228 participants. The flowchart for the study sample is shown in Fig. 1.

In this naturalistic retrospective study, there were in 2003 no planned standard follow-up evaluations. From 2009 onwards, patients treated from 2003 were contacted by mail and asked to complete attached follow-up assessments. After 2017, former patients were invited to a follow-up session at the unit 1–2 years after admission. This design resulted in a large variety in the time span and the types of treatment the participants had received after the initial inpatient treatment. Follow-up was defined as the patient’s latest contact with the ED unit, either by mail or in person, at which the standard questionnaires were completed.

The participants’ written informed consent forms were provided in accordance with the Declaration of Helsinki. The study was approved by the Regional Committee for Medical and Health Ethics for Central Norway (ID: 2009/1864) and the Data Access Committee at Health Trust Nord-Trøndelag (ID: 2023/4505).

### Assessment

Diagnostic assessments were completed at admission by licensed psychologists and medical doctors. All preliminary ED diagnoses were reevaluated at team meetings to which at least two specialists attended. As described by Danielsen et al. [2], the diagnoses were in accordance with criteria of the *Diagnostic and Statistical Manual of Mental Disorders* (DSM-5) [24]. However, for patients included before the release of the DSM-5 in 2013, diagnoses were originally based on the criteria from the Diagnostic and Statistical Manual of Mental Disorders (DSM-IV) [25] and later converted by two ED specialists (one clinical psychologist and one psychiatrist) in accordance with the DSM-5 criteria. Comorbid diagnoses were routinely assessed according to the unit’s procedures and classified according to the diagnostic criteria (see Table 1).

**Table 1:** Baseline characteristics of the complete sample and in diagnostic groups

	Complete sample *N* = 228 Mean (SD)	AN *n* = 140 Mean (SD)	BN *n* = 48 Mean (SD)	BED *n* = 9 Mean (SD)	OSFED *n* = 31 Mean (SD)
Age (years) Range	24.6 (7.9) 16.0-59.6	23.7 (7.8) 16.0-59.6	25.0 (7.2) 17.4–49.1	31.6 (10.3) 22.0-49.5	26.0 (7.9) 17.1–46.7
BMI Range	18.9 (6.8) 10.8–63.3	15.7 (1.8) 10.8–18.5	22.8 (4.4) 17.5–35.9	43.9 (11.7) 30.7–63.3	20.5 (2.4) 17.6–27.7
Duration of illness (years) Range	7.9 (7.3) 1.0–44.0	6.5 (6.8) 1.0–44.0	9.0 (7.3) 2.0–34.0	16.1 (7.1) 7.0–27.0	10.2 (7.3) 1.0–28.0
	n (%)	n (%)	n (%)	n (%)	n (%)
Current depression diagnosis (DSM-5)	114 (50.0)	63 (45.0)	28 (58.3)	4 (44.4)	19 (61.3)
Current anxiety diagnosis DSM-5)	30 (13.2)	18 (12.9)	7 (14.6)	2 (22.2)	3 (9.7)

Note: Duration of illness is self-reported

Abbreviations: BMI, body mass index; AN, anorexia nervosa; BN, bulimia nervosa; BED, binge eating disorder; OSFED, other specified feeding or eating disorder; DSM-5, the diagnostic and statistical manual of mental disorders

At admission, 61.4% (*n* = 140) of the sample were diagnosed with AN (BMI ≤ 18.5). Among these, 70.7% (*n* = 99) had a restrictive subtype, and 29.3% (*n* = 41) a bulimic subtype. A total of 21.1% (*n* = 48) were diagnosed with BN, 3.9% (*n* = 9) with binge eating disorder (BED), and 13.6% (*n* = 31) with other specified feeding or eating disorders (OSFED). Unfortunately, structural assessment of PTSD has not been part of the regular routine at the unit.

### The treatment facility and treatment program

The specialist ED unit (Regionalt Kompetansesenter for Spiseforstyrrelser (RKSF)) is a tertiary treatment facility that is part of the regional adult psychiatric services and accepts patients from age 16. The unit primarily provides services to patients residing within the Central Norway Health Region. However, patients from other parts of the country can be referred to inpatient treatment. The hospital inpatient treatment is provided to the patients free of charge as per the Norwegian health care system. All patients admitted to the unit are first enrolled into an introductory week in which they are given the opportunity to familiarize themselves with the unit and the treatment program. If they choose to continue the admission after this week, they will have to sign a treatment contract and agree to the terms of the treatment program, The unit has an interdisciplinary approach, focusing on both mental and physical health, and the treatment schedule includes a combination of individual treatment and therapeutic groups. Accommodation can also be made for patients with comorbid diagnoses such as trauma disorders. The treatment approach at the unit is grounded in psychodynamic theory, but it has gradually included more elements from other therapeutic approaches. For more information on the unit and the treatment program, this has been thoroughly described previously [26, 27].

### Measures

In this study, self-reported questionnaire data from admission and follow-up were analyzed.

#### ED symptoms

The Eating Disorder Inventory (EDI) is a 91-item questionnaire, measuring psychological traits and behaviors associated with EDs. The third edition of the EDI was published in 2004 but was not available in Norwegian until 2015. Therefore, the previous version, the EDI-2 [28], which has been validated in Scandinavian samples [29, 30] was used. The questionnaire was completed by 216 participants at admission and 206 at follow-up. In the analyses, the EDI-2 sum score, the Symptom scale (Drive for thinness, Body dissatisfaction and Bulimia) and the Psychological scale (Ineffectiveness, Social insecurity, Interpersonal distrust, Interoceptive awareness, Impulse regulation, Perfectionism, Asceticism, and Maturity fears) were used. The Cronbach’s alpha coefficients in the EDI-2 sum score, Symptom scale and Psychological scale were 0.95, 0.90, 0.94 respectively at admission and 0.97, 0.96, 0.95 at follow-up.

The Eating Disorder Examination Questionnaire (EDE-Q) [31] is an adaption of the Eating Disorder Examination and includes 28 items measuring ED behavior over the last 28 days, loading on four factors from which a global score can be generated. The EDE-Q was translated and published in Norwegian in 2008 and was implemented at RKSF in 2009. Thus, no EDE-Q data for patients admitted before 2009 exists. Consequently, patients with data from admission-, discharge and/or follow-up before this time will lack the EDE-Q on all or some of the measurement points, and there is therefore a lower number of respondents on this variable than on other variables, with a total of 136 participants completing the questionnaire at admission, and 218 at follow-up. In the analyses, the EDE-Q global score was used, and descriptions of ED behavior from the EDE-Q at follow-up were used in the definition of remission. The Cronbach’s alpha coefficients in the EDE-Q global score were 0.94 and 0.97 at admission and follow-up.

#### Depression

The Beck Depression Inventory (BDI-II) [32] is a 21-item scale designed to measure depressive symptoms. The questionnaire was completed by 219 participants at admission and 223 at follow-up. Participants’ sum scores were included in the analyses. Cronbach’s alpha coefficients were 0.90 and 0.95 at admission and follow-up, respectively.

#### General psychopathology

The Symptom Checklist-90-Revised (SCL-90-R) [33] is a 90-item questionnaire measuring general psychopathology. The questionnaire was completed by 222 participants at admission and 223 at follow-up, and the mean total scores for the participants were included in the analyses. The Cronbach’s alpha coefficients were 0.97 at admission and 0.98 at follow-up.

#### Interpersonal problems

The Circumplex of Interpersonal Problems (CIP) [34] was used to assess interpersonal problems. The CIP (48 items) is an abbreviated version of the Inventory of Interpersonal Problems IIP64 (64 items) and is based on previous inventories [35, 36]. The questionnaire has acceptable psychometric properties [34] and was completed by 220 participants at admission and follow-up and the participants’ mean total scores were used in the analyses. The Cronbach’s alpha coefficients in the total scores were 0.93 and 0.95 at admission and follow-up.

#### Body mass index

At admission, trained milieu therapists at the ED unit measured height and weight, and BMI (kg/m^2^) was calculated. At follow-up, weight was self-reported.

#### Childhood traumatic events

The occurrence of CTE, defined as any sexual-, physical- or emotional abuse (including neglect) and peer victimization, were collected retrospectively from patients’ medical records. Since this is a retrospective study, information on trauma exposure has not been systematically collected for the purpose of this study throughout the period. However, from year 2005 onwards, the Mini-International Neuropsychiatric Interview (M.I.N.I.) [37] including screening for PTSD was routinely used in the unit. In addition, at admission, all patients answered socio-demographic questions, including their experiences with traumatic events. Information from these sources and from the medical records were used in this study. Traumatic events occurring before age 18 and before ED onset were included. In addition, based on a clinical evaluation of the development of symptoms, three cases (1.3%), in which the exposure happened after ED onset and clearly exaggerated the illness, were included. The degree of severity of the traumatic exposure was a clinical evaluation discussed by three ED specialists with broad clinical experience from working with the patient group (SW, SB, and MD) and divided into three severity levels: Severe, moderate to low or no exposure according to several pre-defined criteria introduced by Temes, Magni et al. [38], including patients’ age at occurrence, length of exposure, relation to the offender, the nature of the occurrence(s) pain and crudeness associated with the occurrence(s). However, although we believe that we had sufficient information to conclude on severity levels, we did not have adequate information on all of the variables needed to complete the computation for severity of the trauma exposure for every patient in accordance with Temes et al. [38]. Thus, the criteria were used as guidelines and no computation was performed. As an example of the decisions made, an unwanted sexual event happening only once in adolescence, in which the abuser was a stranger and there was no physical contact, would typically be coded as CSA of low severity, whereas ongoing sexual abuse from early childhood committed by a parent would be coded as severe CSA. No interrater reliability analysis was performed for the degree of severity of trauma experiences. Due to the low number of participants in the CSA and CTE of moderate and low severity, these groups were merged into CSA of moderate to low severity and CTE of moderate to low severity, respectively. The exposure groups used in this study was any CTE (including CSA) and CSA only. One participant did not want to provide information about sexual abuse, and in analyses including CSA data, the sample therfore consists of 227 participants.

### Definition of remission

The significance of applying reliable definitions of remission has been emphasized [1, 39–41]. To operationalize remission in this transdiagnostic sample, we implemented a definition suggested by Bardone-Cone et al. [40] which incorporates psychological elements, ED behavior and a physical component, and based this on EDE-Q national norms [42].

- Remission: EDE-Q global score within one SD of national norms (≤ 2.5), no binge/purge behavior in the last four weeks and BMI ≥ 18.5.
- Partial remission: EDE-Q global score within two SDs of national norms (≤ 3.6), binge/purge behavior less than once per week and BMI ≥ 17.5.
- Poor or no remission: EDE-Q global score above two SDs of national norms (> 3.6), or binge/purge behavior more than once per week or BMI < 17.5.

### Statistical analyses

One-way ANOVA with Bonferroni post hoc test was used to investigate differences in groups at baseline. The differences in questionnaire scores (*dependent variables*) were examined according to severity of childhood trauma separately in the CSA and CTE groups (*factor/independent variable)*. The standardized effect size Eta squared η2 was calculated and accompanied by the 95% CI in significant differences. Paired t-tests were used to examine the change in mean difference in the questionnaire scores from admission to follow-up. Standardized effect sizes were calculated (Cohen’s *d*). To investigate the association between the severity of childhood trauma, measured by CSA or CTE categories, and the degree of remission, we used Spearman’s correlation. Pairwise comparisons of the degree of recovery between CSA and CTE groups were performed using Mann-Whitney U tests. The exact *p*-values are reported. Cronbach’s alpha coefficient was used to assess the degree of internal consistency of the scales. Data from all questionnaires were missing at baseline for six participants and at follow-up for one participant. Moreover, separate questionnaires were missing for several participants at both time points (see the description of the questionnaires above). Complete-case analyses were carried out. Body weight was missing for a total of nine participants at follow-up, and the BMI for these participants could therefore not be determined. *P*-values < 0.05 were considered statistical significance. The analyses were conducted using the Statistical Package for the Social Sciences (SPSS, version 29) (IBM, Armonk, NY).

## Results

The baseline clinical characteristics of the participants are depicted in Table 1. For the complete sample, the average duration of inpatient stay was 134.7 days (SD = 80.5, range 10–501 days). The average follow-up period was 4.1 years (SD = 3.0, range 0.6–14.0 years) after discharge. About one-third had a follow-up period of two years or less 35.5% (*n* = 81), about one-third between two and five years 31.2% (*n* = 71) and 33.3% (*n* = 76) had a follow-up period of five or more years. There were no significant differences between the follow-up periods in the severity groups of CSA (*F*(2, 224) = 1.98 (*p* = 0.14) or CTE (*F*(2, 225) = 1.52 (*p* = 0.22).

Based on the findings from medical records, 33.0% (*n* = 75) of the participants were classified as having experienced severe CSA, and 16.3% (*n* = 37) as having experienced moderate to low CSA. Among participants with any CTE, including CSA, 48.7% (*n* = 111) and 26.8% (*n* = 61) had experienced severe and moderate to low CTE, respectively.

At baseline, the ANOVA analyses showed statistically significant differences in ED symptoms between severity groups (severe; moderate to low; no exposure), measured by the EDI-2 sum score (*p* = 0.01) and symptom scale (*p* = 0.002)) and depression measured by the BDI-II (*p* = 0.02). The Bonferroni post hoc test showed significant group differences between participants with severe CSA and participants without CSA experience (see Table 2a). Patients exposed to moderate to low CSA consistently scored at a level between the two other groups but were not significantly different from either. In the CTE group (See Table 2b), the ANOVA analyses showed statistically significant differences in ED symptoms, EDI-2 sum score (*p* = 0.5), and EDI-2 symptom scale (*p* = 0.008). The Bonferroni post hoc test indicated no statistically significant differences between severity groups of the EDI-2 sum score. In the symptom scale, the Bonferroni post hoc test showed significant differences between the group of high severity CTE and both the moderate to low group (*p* = 0.02) and the group with no CTE (*p* = 0.04).

**Table 2:** Baseline self-report questionnaires in severity groups of childhood sexual abuse and childhood traumatic events

A: CSA data	Severe CSA *n* = 75 (Group A)	Moderate to low CSA *n* = 37 (Group B)	No CSA *n* = 115 (Group C)	ANOVA	Effect size	Bonferroni Post hoc		
Self-report questionnaires	Mean (SD)	Mean (SD)	Mean (SD)	*(df) F*-value *P*-value	Eta squared η2	Between Groups CSA	Mean difference (95% CI)	*P*-value
EDE-Q global score (*n* = 136)	4.3 (1.3)	4.0 (1.6)	4.1 (1.3)	(2, 133) 0.55 0.58	0.008			
EDI-2 sum score (*n* = 216)	118.8 (40.3)	111.8 (43.9)	101.4 (38.9)	(2, 212) 4.71 **0.010**	0.043	A vs. C	18.5 (3.8; 33.2)	**0.008**
EDI-2 symptom scale	43.4 (15.2)	40.4 (15.2)	35.8 (14.0)	(2, 212) 6.60 **0.002**	0.059	A vs. C	7.9 (2.6; 13.2)	**0.001**
EDI-2 psychological scale	74.9 (30.4)	68.8 (32.8)	65.0 (29.3)	(2, 212) 3.32 0.10	0.022			
BDI-II sum score (*n* = 219)	34.1 (10.7)	31.7 (10.6)	29.4 (12.0)	(2, 214) 3.94 **0.02**	0.036	A vs. C	4.8 (0.67; 9.0)	**0.02**
SCL-90-R mean score (*n* = 222)	1.8 (0.6)	1.7 (0.7)	1.5 (0.7)	(2, 218) 2.46 0.09	0.022			
CIP mean score (*n* = 220)	1.6 (0.6)	1.5 (0.7)	1.5 (0.6)	(2, 216) 1.15 0.32	0.011			

B: CTE data	Severe CTE *n* = 111 (Group A)	Moderate to low CTE *n* = 61 (Group B)	No CTE *n* = 56 (Group C)	ANOVA	Effect size	Bonferroni Post hoc		
Self-report questionnaire	Mean (SD)	Mean (SD)	Mean (SD)	*(df) F*-value *P*-value	Eta squared η2	Between groups CTE	Mean difference (95% CI)	*P*-value
EDE-Q global score (*n* = 136)	4.1 (1.4)	4.2 (1.2)	4.0 (1.5)	(2, 134) 0.25 0.78	0.004			
EDI-2 sum score (*n* = 216)	116.0 (42.3)	102.1 (41.2)	102.4 (35.3)	(2, 213) 3.15 0.05	0.029			
EDI-2 symptom scale	42.3 (15.5)	35.9 (13.7)	36.2 (13.8)	(2, 213) 4.99 **0.008**	0.045	A vs. B A vs. C	6.4 (0.6; 12.3) 6.1 (0.2; 12.0)	**0.02** **0.04**
EDI-2 psychological scale	72.6 (31.1)	64.6 (31.8)	65.4 (26.9)	(2, 213) 1.74 0.18	0.016			
BDI-II sum score (*n* = 219)	33.2 (11.4)	30.6 (10.8)	28.7 (11.6)	(2, 215) 2.97 0.054	0.027			
SCL-90-R mean score (*n* = 222)	1.7 (0.7)	1.6 (0.7)	1.5 (0.6)	(2, 219) 2.27 0.11	0.020			
CIP mean score (*n* = 220)	1.6 (0.6)	1.5 (0.6)	1.5 (0.6)	(2, 217) 0.78 0.46	0.007			

Notes: One-way analyses of variance (ANOVA) were performed between severity groups of CTE. Bonferroni post hoc test; only significant relationships reported, and significant *p*-values are written in bold. Effect size, eta squared η2: small effect size = 0.02, medium = 0.13, and large = 0.26

Abbreviations: CSA, childhood sexual abuse; CTE, childhood traumatic events; EDE-Q, eating disorder examination questionnaire: EDI, eating disorder inventory; BDI, beck depression inventory; SCL-90-R, symptom checklist revised; CIP, circumplex of personal problems; CI, confidence interval

Results from the analyses of change (mean difference) in self-report questionnaire scores from first admission to last follow-up data are presented in Table 3. All groups showed significant change on all self-report questionnaires from baseline to follow-up (*p* < 0.001 to *p* < 0.05, *d* = 0.34 to 1.00), except for the CIP mean total score in the group with CSA exposure of moderate to low severity.

**Table 3:** Mean difference of self-report questionnaire between baseline and follow-up in severity groups of childhood trauma

A: CSA data	Severe CSA (*n* = 75)			Moderate to low CSA (*n* = 37)			No CSA (*n* = 115)		
Self-report questionnaire	Mean difference A – F (95% CI)	*p*-value	Cohen’s *d*	Mean difference A – F (95% CI)	*p*-value	Cohen’s *d*	Mean difference A – F (95% CI)	*p*-value	Cohen’s *d*
EDE-Q global score	1.0 (0.5; 1.6)	**< 0.001**	0.57	1.1 (0.0; 2.1)	**0.047**	0.51	1.2 (079; 1.6)	**< 0.001**	0.69
EDI-2 sum score	33.2 (20.5; 46.0)	**< 0.001**	0.64	29.4 (15.3; 43.5)	**< 0.001**	0.78	33.1 (25.1; 41.1)	**< 0.001**	0.83
EDI-2 symptom scale	12.5 (7.6; 17.3)	**< 0.001**	0.65	11.0 (5.2; 16.9)	**< 0.001**	0.70	10.8 (7.5; 14.06)	**< 0.001**	0.65
EDI-2 psychological scale	19.2 (10.6; 27.8)	**< 0.001**	0.55	14.8 (4.7; 24.9)	**0.006**	0.56	21.4 (15.9; 26.9)	**< 0.001**	0.79
BDI-II sum score	9.8 (6.5; 13.2)	**< 0.001**	0.70	13.2 (8.5; 17.9)	**< 0.001**	0.98	11.7 9.2; 14.2)	**< 0.001**	0.89
SCL-90-R mean total score	0.4 (0.2; 0.6)	**< 0.001**	0.51	0.5 (0.3; 0.8)	**< 0.001**	0.74	0.6 (0.5; 0.7)	**< 0.001**	0.86
CIP mean total score	0.2 (0.1; 0.4)	**0.005**	0.34	0.2 (-0.0; 0.4)	0.11	0.28	0.3 (0.2; 0.4)	**< 0.001**	0.49

B: CTE data	Severe CTE (*n* = 111)			Moderate to low CTE (*n* = 61)			No CTE (*n* = 56)		
Self-report questionnaire	Mean difference A – F (95% CI)	*p*-value	Cohen’s *d*	Mean difference A – F (95% CI)	*p*-value	Cohen’s *d*	Mean difference A – F (95% CI)	*p*-value	Cohen’s *d*
EDE-Q global score	0.9 (0.5; 1.4)	**< 0.001**	0.53	1.4 (0.8; 1.9)	**< 0.001**	0.77	1.1 (04.; 1.9)	**0.003**	0.57
EDI-2 sum score	29.0 (19.2; 38.8)	**< 0.001**	0.61	31.2 (20.3; 42.2)	**< 0.001**	0.79	38.7 (26.4; 51.0)	**< 0.001**	0.92
EDI-2 symptom scale	10.8 (7.1; 14.6)	**< 0.001**	0.59	10.5 (6.0; 15.1)	**< 0.001**	0.64	13.0 (8.0; 17.7)	**< 0.001**	0.77
EDI-2 psychological scale	16.9 (10.3; 23.6)	**< 0.001**	0.52	18.4 (10.0 (25.9)	**< 0.001**	0.68	24.7 (16.0; 33.4)	**< 0.001**	0.83
BDI-II sum score	9.6 (6.9; 12.4)	**< 0.001**	0.69	13.3 (9.8; 16.7)	**< 0.001**	1.00	12.3 (8.7; 15.9)	**< 0.001**	0.97
SCL-90-R mean total score	0.4 (0.3; 0.6)	**< 0.001**	0.57	0.6 (0.4; 1.9)	**< 0.001**	0.86	0.6 (0.4; 0.8)	**< 0.001**	0.82
CIP mean total score	0.2 (0.1; 0.3)	**< 0.001**	0.34	0.2 (0.0; 0.4)	**0.02**	0.35	0.3 (0.2; 0.5)	**< 0.001**	0.62

Note: Paired *t*-tests have been performed between self-report questionnaires scores at admission and follow-up in severity groups of CSA and CTE. Significant *p*-values are written in bold

Abbreviations: CSA, childhood sexual abuse; CTE, childhood traumatic events; A: admission. F: follow-up. CI, confidence interval; EDE-Q, eating disorder examination questionnaire; EDI, eating disorder inventory; BDI, beck depression inventory; SCL, symptom check list; CIP, circumplex of personal inventory

The classification of remission groups was based on the last complete follow-up data for each participant. The classification was accomplished in accordance with the defined remission criteria, and for 217 participants categorized with CSA and for 218 participants categorized with CTE. The classification could not be accomplished in ten participants due to missing data. One participant did not share information about CSA and is therefore also missing in the remission classification. The follow-up period covered a large range, from 0.6 to 14 years.

Data for remission in each of the CSA and CTE groups are summarized in Table 4. A lower percentage of those with severe CSA were in remission than among those with moderate to low or no CSA. However, there was no statistically significant correlation between CSA severity and remission (Spearman’s correlation − 0.09, *p* = 0.175).). There was a statistically significant association between CTE severity and remission (Spearman’s correlation − 0.15, *p* = 0.032). Pairwise comparisons indicate that this association is explained by a difference in remission rate between the group with high severity CTE and the group with moderate to low severity CTE (*p* = 0.051) and between the group with high severity and no CTE exposure (*p* = 0.067). There is no evidence of a difference between the group with moderate to low severity and no CTE (*p* = 0.970).

**Table 4:** Remission at follow up in severity groups of child sexual abuse and childhood traumatic events

	Data based on hospital records						
	Childhood sexual abuse (CSA) (n = 217)				Childhood traumatic events (CTE) (n = 218)		
	Severe CSA *n* = 72	Moderate to low CSA *n* = 36	No CSA *n* = 109		Severe CTE *n* = 104	Moderate tor low CTE *n* = 61	No CTE *n* = 53
	n (%)	n (%)	n (%)		n (%)	n (%)	n (%)
Remission	19 (26.4)	13 (36.1)	38 (34.9)		25 (24.0)	24 (39.3)	21 (39.6)
Partial remission	13 (18.1)	6 (16.7)	22 (20.2)		21 (20.2)	11 (18.0)	9 (17.0)
Poor or no remission	40 (55.6)	17 (47.2)	49 (45.0)		58 (55.8)	26 (42.6)	23 (43.4)

## Discussion

### Prevalence of childhood trauma exposure

The first aim was to investigate the frequency of CSA and/or CTE in a transdiagnostic group of female patients with ED that had previously been treated in an inpatient unit. Analyses showed that a total of 49.3% of the participants had been exposed to CSA, while 75.5% had been exposed to any CTE including CSA. These numbers were higher than those found by Molendijk et al. [5] in a review and meta-analysis of published studies on CM and EDs. However, it is important to note that the aforementioned paper was based on 82 studies which predominantly investigated out-patients. By distinguishing between in- and outpatients, the same authors found that the prevalence rates among these were 45% vs. 29% respectively [5]. The patients in the current sample were inpatient at a tertiary treatment facility, and it is therefore expected that they had elevated events even compared to other inpatient samples. Interestingly, the frequencies from our study align with those from another tertiary ED unit in Norway, in which it was found that 38.7% had been exposed to CSA and 71.0% to any CM [6]. The difference in frequency between the current study and previous studies might be caused by differences in how data on trauma exposure was collected, and by the fact that we chose to include peer victimization in our definition of CTE. Based on clinical knowledge, including this category as a CTE is perceived as meaningful since bullying are events that patients themselves report as significant in their childhood and in the development of their ED. In addition, the importance of peer victimization/bullying was underlined by Lie et al. [14] who found elevated rates of bullying in patients with ED, especially among patients with binge-eating/purging. In the same study, patients that had been exposed to bullying had more thoughts of self-harm and suicide, indicating the severe effect of such events. Moreover, Idsoe, Vaillancourt et al. [43] argue for defining bullying as a repetitive interpersonal trauma, which most often is recurrent and ongoing, and in a literature review and meta-analysis, there is support for an association between bullying and PTSD [44].

Notwithstanding these factors and in accordance with previous research, findings from the present study clearly demonstrate the high frequency of CSA and CTE also in a transdiagnostic sample with severe EDs in need of inpatient treatment and underscore the need to assess trauma events in patients, and to address these in therapy.

### Baseline characteristics differences

Our second aim was to investigate the difference in baseline symptom scores between patients with EDs with CSA and/or CTE events of different severity and patients without such events. Findings showed that patients exposed to severe CSA reported more difficulties on two of three measures from the EDI-2 and on the BDI-II compared with patients with no CSA exposure. Likewise, patients exposed to severe CTE reported more difficulties on the EDI-2 symptom scale than the group with no CTE exposure, but also higher than the group reporting moderate to low exposure. This is in line with previous research finding a dose-response relationship in patients with EDs [5, 16]. Moreover, a dose-response effect has also been found between exposure to CTE and the occurrence of any psychiatric disorder in a large study on adolescence [45]. Furthermore, in a systematic review and meta-analysis, individuals that had experienced four or more traumatic events during childhood had an increased risk of several health problems in adulthood [46]. An accumulative effect of trauma exposure with higher levels of purging behavior in individuals that had trauma events both in childhood and adulthood compared to those who had experienced trauma in either childhood or adulthood has also been observed [47].

### Treatment outcome differences

The last aim was to investigate changes in symptoms from baseline to last follow-up and rates of remission at follow-up. At follow-up, all groups showed significant improvement on all self-report measures from the EDE-Q, the EDI-2, the BDI-II and the SCL-90. This indicates that patients in all groups experienced a decrease in symptoms between baseline and at follow-up at an average of 4.1 years post treatment initiation, and thus that patients with EDs and trauma improved from baseline to follow-up. The results on the CIP, however, are more mixed. Notably, the effect sizes are generally smaller for the change from baseline to follow-up on this measure, and especially so in the CSA group, indicating that changes in interpersonal functioning seem to change less over time compared with the other self-report measures. This may suggest that such problems are difficult to change, and that interpersonal difficulties may result from coping mechanisms resulting from early maladaptive schemes (for instance avoiding others due to low self-esteem) in patients with EDs and trauma exposure, as suggested by Aloi, Rania et al. [48] or represent partly stable personality traits.

There was evidence of an association between severity of CTE and remission group affiliation. Although not statistically significant, there was also an indication of a similar association between severity of CSA and remission group affiliation, with a lower percentage of patients with severe CSA being in the remission group and a higher percentage being in the poor or no remission group than in the moderate to low or no CSA groups. Since CSA experiences was also included within the CTE group, these results partly support the findings from Eielsen et al. [6] who also investigated a transdiagnostic sample, but are different than the findings from Castellini et al. [19], who found high levels of remission from the ED both in patients with childhood abuse (66.7%) and without childhood abuse (59.0%) at 3-year follow-up. However, in the aforementioned study, a larger proportion of patients with ED and trauma exposure exhibited remission from the ED but still fulfilled the criteria for one or more comorbid disorders (54.6%) vs. (28.0%) in the ED with trauma and the ED without trauma group, respectively [19]. Again, this difference might be attributable to different samples, inclusion- and exclusion criteria.

### Strengths and limitations

The strength of this study is the inclusion of a relatively large sample, the thorough diagnostic process, and the length of the follow-up period. An additional strength is the inclusion of neglect among the CTEs, since knowledge about the effect of this variable is lacking in the child maltreatment field [49]. However, as this is a naturalistic retrospective clinical study, it also has some limitations. First and foremost, the rate of eligible patients that did not participate in the study (due to death, lack of consent, lack of invitation or lack of response to invitation), was relatively high at 45.2%. Unfortunately, due to Norwegian Health Research legislation, we do not have the opportunity to investigate if there were any systematic differences at baseline between the participants and the non-participants, but there is a chance that a self-selection bias may have occurred.

Moreover, as mentioned, since the EDE-Q was not translated into Norwegian until 2008, only 136 patients completed this questionnaire at baseline (218 at follow-up), so the findings from baseline on this questionnaire must be interpreted with caution. Furthermore, since the information about patients’ experience of CSA/CTE was collected from medical records, no formal structural assessment of trauma events was done as part of this study, and although the evaluation of the severity of these events was conducted based on a set of predefined criteria, there was also an aspect of clinical judgement that might have been affected by the clinicians doing this evaluation. In addition, although the severity of the described events were rated, it is important to note that it is the level to which the person experiences an event as traumatic that is most important, not the severity of the incident in itself [50]. These issues might contribute to limiting the validity and reliability of the findings. Additionally, to investigate the long-term changes in symptoms for these patients, we wanted to get as long a follow-up period as possible. Therefore, for the purpose of this study, the very last complete follow-up was chosen. Since this is a naturalistic study, the patients would have had a vastly different number of follow-ups during this period, they would have sought different levels of therapy and they would had different length of follow-up, which might have affected the results.

Furthermore, since the patients were all inpatients at a tertiary treatment facility, findings might not be applicable to the ED population at large. In addition, there was no formal structural diagnostic assessment of PTSD for the entire sample. The importance of PTSD should be investigated in future studies. Moreover, as described, including peer victimization as a category of CTE is clinically and theoretically relevant, however, we acknowledge that there is a possibility that incidents that most people experience during childhood have been included as traumatic events. In addition, the evaluation of exposure of traumatic events did not include an evaluation of timing of exposure or repetition of traumatic events happening after age 18 or after the onset of the ED. This fact might have masked interesting findings since trauma exposure in childhood combined with re-traumatization in adulthood has shown to increase the severity of symptoms [47], and might also have an impact on the outcome. Furthermore, although repeated exposure was defined to indicate greater severity, an evaluation of dose-response was not formally conducted. In addition, research has shown that the association between trauma and ED behavior may be strongest in EDs with binging behavior [5, 16], and due to the transdiagnostic nature of the current study, no analyses were performed on outcome in the different diagnostic groups or in patients with or without binge/ purge behavior. Lastly, remission was defined based on ED measures only and did not take trauma symptoms into account. However, the findings from this study show that in a heterogenic patient group with different types of EDs, those with comorbid CTE have more symptoms and a less favorable outcome.

## Conclusions

We found that patients with EDs had a high prevalence of CSA and CTE and that patients with EDs and trauma experiences displayed more symptoms at baseline. Furthermore, although all groups showed decreased levels of symptoms from admission to follow-up, there was an association between severity of CTE exposure and remission. These findings point to the importance of including a broad assessment of CTE, including an evaluation of severity in patients at treatment initiation, and to take this comorbidity into account in treatment planning when present.

## Data Availability

The data used in this study are not publicly available due to conditions for consent from participants.

## Abbreviations

AN: Anorexia nervosa
ANOVA: Analyses of variance
BDI: Beck depression inventory
BED: Binge eating disorder
BMI: Body mass index
BN: Bulimia nervosa
CBT: Cognitive behavioral therapy
CIP: Circumplex of interpersonal problems
CM: Childhood maltreatment
CTE: Childhood traumatic events
CSA: Childhood sexual abuse
DSM: Diagnostic and statistical manual of mental disorders
ED: Eating disorder
EDE-Q: Eating disorder examination questionnaire
EDI: Eating disorder inventory
IIP: Inventory of interpersonal problems
OR: Odds ratio
OSFED: Other specified feeding or eating disorder
PTSD: Post-traumatic stress disorder
RKSF: Regionalt kompetansesenter for spiseforstyrrelser
SCL: Symptom checklist
SD: Standard deviation
SPSS: Statistical package for the social sciences
